# Comment on Lachenmeier *et al* (2020) “Are side effects of cannabidiol (CBD) products caused by tetrahydrocannabinol (THC) contamination?”: disputation on various points in the publication

**DOI:** 10.12688/f1000research.25354.1

**Published:** 2020-08-04

**Authors:** Daniel Kruse, Bernhard Beitzke

**Affiliations:** 1 European Industrial Hemp Association (EIHA), Hürth, NRW, 50354, Germany

**Keywords:** Cannabidiol, tetrahydrocannabinol, hemp, illegality, daily intake, toxicity, side effects

## Abstract

This Correspondence article is a counterstatement to a Brief Report published by Lachenmeier and co-workers on 17th February 2020 in F1000Research: “Are side effects of cannabidiol (CBD) products caused by tetrahydrocannabinol (THC) contamination?”. This counterstatement proposes that the authors of that article neither present proof or evidence for the alleged side effects of CBD products (no case reports presented with utilisable data), nor do they show that side effects are due to the presence of THC.

Primarily, there is no clear definition of THC because the authors do not explain whether they mean Delta9-THC only (without its precursor tetrahydrocannabinolic acid (THCA)) or total-THC (the sum of Delta9-THC and its precursor THCA, normalised to THC); indeed EU Recommendation 2016/2115 on the monitoring of cannabinoids in food requires the measurement and documentation of the precursor acids complementary to the decarboxylated cannabinoids. The key part of the authors’ work – Table 2 with the assessment of the CBD products – leaves the reader in the dark about the nature of “THC”. This is all the more concerning because acid-free Delta9-THC is psychotropic but THCA is not.

Additionally, the classification of the CBD products (“toxicity assessment”) presented is based on the assignment of the quantitative relation to the LOAEL (lowest observed adverse effect level) of THC (2.5 mg of acid-free Delta9-THC per adult and day as assigned by EFSA, 2015). However, many assumptions by Lachenmeier *et al.* on daily intake of CBD products are questionable, in particular food supplements, where the recommended daily consumption was missing on the label.

Finally, the authors of the paper also compare their findings with the German recommendations on maximum levels of total-THC in food, ignoring that those limits refer to total-THC and the ready-to-eat products, and not to the food ingredient itself – in particular hemp tea products.

## Introduction

This Correspondence article was written by members of the European Industrial Hemp Association (EIHA; https://eiha.org/) and EIHA Advisory Committee to highlight their concerns about the Brief Report published by Lachenmeier *et al.* ^1^ on 17th February 2020 in F1000Research: “Are side effects of cannabidiol (CBD) products caused by tetrahydrocannabinol (THC) contamination?”. The content and argumentation of the Brief Report has alienated our organization in many ways. For this reason, as a European professional association, we feel compelled to comment on this publication and its content.

The article by Lachenmeier *et al.* revealed the following:

- purported THC-like side effects aimed to show of CBD or CBD-containing products cannot be sequelae of CBD conversion to THC by gastric fluid but are the effects of the residual THC in such products;
- CBD-containing hemp products (teas, extracts sold as food supplements) give rise to THC-like side effects because in many cases either the LOAEL or the European Acute Reference Dose of THC will be exceeded after consumption of such products;
- the high THC-content of CBD products is a “scandal on the food market”, and food business operators “placed unsafe and approved products on the market”;
- the current regulatory framework were “insufficient to adequately regulate products in the grey area between medicines and food supplements”.

## Alleged side effects of CBD products

Firstly, it should be noted that, as the authors themselves admit, cases of alleged side effects of products containing CBD claimed in the publication are anecdotal. It is striking that no reference is given as proof of evidence. Instead, the authors try to present findings on effects of Epidiolex® as evidence for their statement on CBD-containing hemp products. Indeed, the reference to rare side effects of the Epidiolex® studies is displaced, especially since Epidiolex® (against special forms of children's epilepsy) is "pure" CBD with a much higher dosing regimen for a pharmaceutical/metabolic action and cannot be compared with low dosed CBD oils and hemp extracts with naturally present levels of Cannabinoids. On the contrary, it has been suggested that some adverse effects of CBD in the clinical studies (for Epidiolex®) may relate to interactions with other antiepileptic drugs ^2^. Moreover, an assessment of the drug status of Epidiolex® by the US Public Health Service came to the conclusion: “ *Thus, it is unlikely that THC contributed to the slight positive responses on some of the subjective measures or contributed to the euphoric [adverse event] responses reported following the higher doses of CBD.” ^3^*

Hence the question is: have the purported side effects after uptake of CBD oils or hemp extracts been measured and proven? Have there been serious side effects showing that these products were not safe, and which products exactly gave rise to the reported side-effects?

All this is not explained in the article by Lachenmeier *et al*, although the title of this publication suggests that these side effects are hard facts. No reference or proof is given for the alleged side effects of the CBD products in question. Furthermore, a statement by the British FSA ( *FSA 20-01-03*) ^4^ states:

*"18. We have not been made aware of any safety incidents relating to CBD products on the market, so we are not planning to insist on an immediate removal of the products from the shelves. That said, it is important that industry puts these products through the authorisation process as the process is there ..."*

This statement is representative for CBD products all over Europe because the UK was and is an important market for the Food Business Operators (FBOs) on the European continent.

## "THC" definition and estimation of daily dose of products

The main subject of the article by Lachenmeier *et al* is "THC”, which is neither precisely defined in the publication nor correctly explained, i.e. there is no differentiation between Delta9-THC (acid-free) and Delta9-tetrahydrocannabinolic acid (THCA), the latter being the non-psychotropic precursor of THC and not contributing to the *"toxic effects"* of "THC" ^5^. From the raw data published in Table 2 of Lachenmeier *et al*, the type of THC cannot be defined for each case, i.e. whether this should mean total-THC (which is the sum of Delta9-THCA, corrected by the molecular weight factor, and Delta9-THC) or Delta9-THC (acid-free) only.

Pursuant to the EU Recommendation 2016/2115 ^6^ on the monitoring of cannabinoids in food, THC should be differentiated correctly between Delta9-THC and its precursor THCA; their respective concentrations should be measured separately, and the contents of both compounds documented correctly, in particular in a scientific paper.

In favour of the authors, we will assume here that the "THC" measured and mentioned in the publication refers only to Delta9-THC (acid-free), and that the analytical method used for this purpose is able to quantify the latter separately from THCA ( *note:* natural products that have not been heated always contain a part as THCA) ^7^. This is also relevant for the samples containing cannabidiolic acid (CBDA) and/or cannabigerolic acid (CBGA) (5 samples tested), as most probably the main part of their "THC"-content consists of THCA, which should not be included in the toxicity assessment based on the Delta9-THC content.

Moreover, the statement in Lachenmeier *et al. "Out of 67 samples, 17 samples (25% of the collective) were exceeding the THC LOAEL [Lowest Observed Adverse Effect Level] and were assessed as harmful to human health…"* is not scientifically correct and cannot be assumed as hard fact. The "THC" level is in fact the result of the measurement of the "THC" concentration by analysis, while the LOAEL is the lowest amount of a substance, which, when measuring certain effects in animal or human studies, shows just observable adverse effect(s). The missing link between the result from the analysis and the dictum of " *LOAEL has been exceeded*" is the (recommended) daily dose of the specific product together with its measured THC-concentration. These data are only available in the raw data published by Lachenmeier *et al.* ^8^ which should be shown in the publication itself. It should also be noted that for those products for which a recommended daily dose was missing on the label, the authors made an estimation of these daily doses. In many cases, however, they assumed a very high daily dose, which in our view is far from being practical, as can be illustrated by the following examples:

****Tea products (hemp flowers or leaves):**** the authors base their calculation on a *"probable intake of 2 portions/day"* (8 g of tea leaves or flowers per person daily), or they assume a daily consumption for tea products taking into account a worst case scenario, concluding the " *LOAEL **may be** exceeded*". However, in Table 2 it is stated that the THC-LOAEL **is** exceeded (marked in red). This is confusing to readers who will be unable to distinguish which statement is correct.

Furthermore, the THC intake for all tea products is calculated by multiplication of the estimated daily portion with the measured THC-content in the hemp tea leaves, hence the authors insinuate the ingestion of 100% of the THC found in the hemp “tea leaves”. This type of calculation is not correct. Firstly, a 100% carry-over of THC into the tea preparation (infusion) has been refuted in the scientific literature ^9^. Secondly, as to the German guidelines on total-THC-content in foodstuff, the THC guidance value for tea beverages refers to the ready-to-eat or ready-to-drink products and not to the hemp “tea leaves” green matter. Among EIHA members, there are FBO's who have been testing hemp leaf infusions in water for years, and these reports are available ^10^. This type of estimation may lead FBOs to wonder why they recommend a daily dose on the label as well as a brewing instruction, if this is then ignored or overruled by an authority in the evaluation of marketability.

****Syrup with hemp flower extract:**** a daily portion of 130 g is assumed (because of a missing recommended dose), thus exceeding European Food Safety Authority (EFSA)'s Acute Reference Dose (ARfD) ^11^. One third of this amount (43 g/d) would usually be an extremely high daily intake of syrup for human consumption in everyday practice and would result in a THC intake below the ARfD (approx. 56 µg/d). This would not be admonished, for example in Austria ^12^. The assignment of a daily dose of 130 g therefore seems to have been arbitrary and possibly results-oriented only.

***Cannabis shot*:** a daily dose of 60 g is estimated and assumed, which even for a food supplement is objectively to be regarded as very exceptional and extremely high; this amount results in a THC uptake of only 8 µg/d. This product is admonished by Lachenmeier *et al.* for exceeding the German guidance for THC-levels in food, even though they are actually not regulated by law let alone made binding. In addition, the German recommendations apply to total-THC (including THC acids) and only for ready-to-eat products (i.e. those which the consumer finally ingests after preparation).

***CBD oil:*** first line in Table 2, the following assumption was made (see footnote 2) about this product: *"No labelling about dosage provided on the label. For this reason the consumption of the whole bottle at once as worst case exposure scenario was assumed."*. In all objectivity, this is exaggerated and not a realistic scenario. First, we can assume that it is well known to the consumer of a food supplement on how to take it. Therefore, the consumer will not take the contents of the entire container or bottle at once. The typical consumer of a food supplement is most likely a well-informed person proactively following a healthy lifestyle, feeling responsible for their own health, and who is therefore willing to purchase products that will help to maintain and to strengthen homeostasis – to stay healthy for a longer lifespan. Second, a food supplement is not a basic food (see legal definition of "food supplements" in Art. 2 lit. a of Regulation 2002/46/EC ^13^). Third, if there are food supplements with “CBD oil” on the market without correct labelling (e.g. without a recommended daily intake) this constitutes the wrongdoing of some individual market players and not of the whole industry.

For some samples with the recommended daily dose missing on the product label (marked with footnote 4, Table 2, in Lachenmeier *et al.*), the basis for the toxicity assessment is unclear from Table 2, whereas in the raw data there is a daily portion given (e.g. 5.0 g for the “CBD buds” or 8.0 g for “Hemp tea with flowers”) and this portion is used for the daily THC-intake calculation.

Considering these examples, the data in Table 2, on which the publication is based, are derived from unclear premises on the nature of "THC" and the daily dosing of the CBD-containing products. Many parts are arbitrarily based on assumptions that are out of touch with reality, and which we do not consider justified.

Admittedly, a dosage or recommended daily intake should always be found on the label of food supplements (see Article 8(2) of Regulation 2002/46/EC). The recommended intake that consumers usually find on the packaging of the products should be clear and concise, and it should be explicitly indicated “not to exceed the recommended dose” and "food supplements should not be used as a substitute for a healthy and varied diet". However, the many cases in which the respective manufacturers have done this correctly seem to be dismissed by the authors.

## Assessment of the impact of CBD products

The assumption stated in the publication by Lachenmeier *et al.* that *"the adverse effects of commercial CBD-products"* (still to be proven by evidence) *"are based on a low dose effect of THC and not due to effects of CBD itself"* lacks sufficient scientific and comprehensible justification as this is only based on the known toxicological effects of isolated Delta9-THC (precisely synthetic Delta9-THC). The interaction between Delta9-THC and CBD, which has been investigated in many studies, is not taken into account here by the authors. Obviously, however, the scientific community is aware that the effects of “THC” are indeed mitigated by CBD (see e.g. Zuardi *et al.,* 1982 ^14^). A more recent publication on "Lower-Risk Cannabis" ^15^ even recommends with the note: *"Given the evidence of CBD’s attenuating effects on some THC-related outcomes, it is advisable to use cannabis containing high CBD:THC ratios. Evidence Grade: Substantial.".* The mechanisms of this interaction are not fully elucidated yet, however, currently it is acknowledged that CBD is a negative allosteric modulator of the CB1 receptor ^16^. It is surprising that the authors do not mention these well-known scientific findings.

## Alleged "illegality" of all hemp products containing CBD

The authors further claim: *"Basically all available CBD products based on hemp extract marketed as food or food supplement within the EU are therefore illegally sold".* Although this is a regulatory issue, we think it is necessary to comment on this blanket statement.

A general statement on the non-conformity of all available CBD-products with EU regulations is, in our opinion, outside of the scope of the publication. As stated already many times by jurisdiction, the assessment, for example on compliance with the Novel Food Regulation (EU), is always a case-to-case evaluation by authorities or at court. It must also be emphasized that the European Novel Food Catalogue is legally not binding (see legal disclaimer on the corresponding website of the EU COM ^17^). For court decisions, the entries in the NF Catalogue are just indicators or circumstantial/presumptive evidence. Moreover, the burden of proof for the novelty of a food is up to the person or body who makes this statement, whereas the economic operator(s) bear(s) a so-called secondary burden of proof, if necessary, which, however, must not lead to the reversal of the burden of proof ^18^. Although the Federal Administrative Court in Germany (BVerwG) has not yet had to make an explicit statement on this legal issue, in a decision on 29 January 2010 it already indicated that it has doubts about the burden of proof regulation, which some administrative courts have practised elsewhere to date ^19^.

Since experience shows that the federal courts in Germany coordinate their case-law on one and the same provision among themselves, it can be assumed that the BVerwG will ultimately follow the aforementioned legal interpretation of the Federal Court of Justice. This is also in view of the fact that the European Court of Justice (ECJ) already imposes the burden of proof on the party claiming the existence of a pharmacological effect of a foodstuff in cases of doubt. The BVerwG has already fully endorsed this legal interpretation of the ECJ on the rule on the burden of proof there ^20^.

Hence, the ECJ did not impose the burden of proof of the non-existence of a pharmacological effect of a foodstuff on the FBO concerned. Therefore, it is all the more unlikely that the ECJ now should come to a different rule on the burden of proof in the issue of a possible "novelty" within the meaning of the Novel Food Regulation and an associated abstract health risk, according to which the FBO were required to prove the non-existence of a "novelty". This would be completely contrary to the legal doctrine as well as to the factual system.

What an authority can do, of course, is to conclude within its expertise on the non-marketability of a specific product because it is "unsafe" for the consumer within the meaning of Article 14(1) and (2) of Regulation (EC) No 178/2002 ^21^, i.e. injurious to health or unfit for human consumption (and this is the primary realm of the local authority). However, such decisions must also be based on scientific evidence (cf. Article 3 et seq. of Regulation (EC) No 178/2002).

## Value judgement on food producers of hemp products

Lachenmeier *et al.* also claim that *"In our opinion the systematically high THC content of CBD products is clearly a “scandal” on the food market. Obviously, the manufacturers have – deliberately or in complete ignorance of the legal situation – placed unsafe and unapproved products on the market and thus exposed the consumer to an actually avoidable risk."*

We consider this sweeping and polemical value judgement misplaced in a scientific work. Reputable, professional producers of food and food supplements always ensure the quality and safety of their products in all steps of production and also observe legal compliance with European and national regulations. These producers have a well-established quality assurance and control for their products and ensure correct labelling, in particular for correct consumer information with a recommendation on the daily intake of the product.

In fact, the legal situation is not as clear as purported by the authors, and it cannot be generally stated that all CBD-containing products are Novel Foods under the European Novel Food Regulation. At the end of 1997, even the EU Commission with its Standing 2015/2283 Committee on Foodstuffs had established and confirmed that foods that contain parts of the hemp plant, such as hemp flowers, are not covered by Regulation (EC) No. 258/97 on Novel Foods and Novel Food ingredients ^22^

In Germany, the Federal Government publicly declared the following in the German Bundestag: *“The opinions of the European Commission confirming that foods containing parts of the hemp plant are not novel foods remain valid. However, it cannot be concluded from them that all products of the hemp plant, including isolated individual substances such as cannabinoids or extracts enriched with cannabinoids, would be marketable as food. In addition … it must always be checked whether a product in its composition was used as a foodstuff to any significant extent in the EU before 15 May 1997. Otherwise, as in the case of cannabinoids, it must be considered as a novel food” ^23^ .*

The EIHA has already presented evidence to the European Commission, according to which hemp extracts have been consumed as food for centuries, and that the so-called "low-THC" varieties from *Cannabis sativa* L. (thus “industrial hemp” or “usefulness hemp”) have always contained CBD. At this point, it should be mentioned that especially in these industrial hemp varieties – including those that were listed in the EU catalogue of varieties long before 1997 – the relevant CBD content in relation to THC is very high in comparison to "high-THC" cannabis varieties. Therefore, such hemp extracts (e.g. those not enriched with CBD) are traditional food and will not fall under the Novel Food Regulation.

Hence, the statement by Lachenmeier *et al.* that the products are unsafe is another blanket statement and must refer to products only that have been in fact proven unsafe for consumption according to current requirements. However, this publication does not scientifically verify this and presents no proof. Moreover, it has been decided many times by jurisdiction that the decision on the novelty of a food is always based on a case-to-case evaluation of the special product in question.

Admittedly, there are always so-called "cowboys" or "black sheep" in the market – as in every industry – disregarding the regulations, trying to make a quick profit with the hype around "legalisation of cannabis" and cannabinoids. This phenomenon is unfortunately also evident in cases of questionable quality and insufficient attention to labelling (e.g. missing recommended intake).

However, discrimination can also occur from undifferentiated action of the authorities with regard to quality and the legal situation vis-à-vis respectable market participants, which clearly leads to abstruse marketing strategies of so called "free riders". Such companies, for example sell plain hemp seed oil through pharmacies at exorbitant prices and suggest to the consumer that it is a particularly effective drug or food supplement ^24^. This situation is also EIHA's concern because this jeopardises the reputation of the hemp industry. However, EIHA, as an association of voluntary members of farmers and producers, can only exert positive influence on its own members, but cannot discipline other unwilling market participants. Therefore, EIHA cannot itself control the market, but could help the authorities to regulate it in a scientifically sound, safe and reasonable way – subject to constructive dialogs and cooperation. EIHA therefore demands clear industry standards that are mandatory for all FBO’s in the sector to ensure compliance with the law, consumer safety and also legal certainty for businesses. Work is underway to harmonise the "self-regulated" quality standards of some national interest groups or individual economic operators. The time spent by EIHA and its members as companies in defending themselves against the arbitrariness of individual authorities could be used more efficiently and constructively in mutual dialogue. Unfortunately, earlier offers of exchange and cooperation in Germany, unlike in other European countries, have so far been largely ignored. This should also be mentioned against the background that in the meantime EIHA has decided to carry out extensive toxicology studies on CBD and THC conducted by a third party ^25^.

## Judgement of the hemp industry in the food sector

In Lachenmeier *et al.*’s conclusion, the authors quote that *"Currently CBD users must be aware that they may be ‘participating in one of the largest uncontrolled clinical trials in history’”.* EIHA does not accept such an insinuation.

The quote ‘participating in one of the largest uncontrolled clinical trials in history’ is from a Newsweek article calling for attention to a new supposed scandal (see ref 33 ^26^ in Lachenmeier *et al.*), and is stated by Pal Pacher, an investigator at the National Institutes of Health in the US. It should be noted that this is his own personal opinion and also refers to the situation in the US and not in Europe. In the US, there are different regulations and THC limits for products. It is not known at all to which products with which THC-contents Mr Pacher refers to in his statement. However, since Lachenmeier *et al.* use this quote in their publication, they are effectively transferring this statement to the whole European hemp industry without any critical review to the situation in Germany and Europe. This statement is not supported by any evidence of accordingly serious incidents or poisoning, which can be attributed to the intake of these so-called "unsafe" products (see the UK FSA statement cited (footnote 4)) in Germany or the EU. So, where is the evidence for the purported side effects and products being unsafe?

Notably, EIHA's members and other professional market operators would like to iterate that they do not use their customers as human guinea pigs.

## Proposal for a legal ban on hemp extracts

Finally, the authors of the publication suggest that *"products based on hemp extract with a similar composition could be treated as illegal narcotics, prescription drugs or novel foods in order to resolve conflicting rules in the field of narcotics, drugs and food law".* We feel that this statement is unsubstantiated.

It is well known that the legal classification of the products offered on the market depends on the composition of the products, on the way they are processed and, above all and primarily, on their objective intended use! Of course, products containing cannabinoids can – depending on their objective intended use – be used for food, food supplements, cosmetic products or as pharmaceuticals as well. It is known and common knowledge that certain food ingredients and substances may be legally sold both as food supplements and as pharmaceuticals, as their use and declaration depends mainly on the dosage of the active ingredient(s) and the method of administration, e.g. the route of administration (oral or intravenous or other) and of course depends on the intended and declared use.

One of many examples is melatonin, where the low-dose substance is sold as a food supplement and the higher-dose form can be sold as a medicinal product (this is the case in Germany at least). Another example is garlic, which is sold as a food, food supplement and pharmaceutical, depending on its use and dosage form. In this case, the ECJ only a few years ago decided exactly on this juxtaposition of food (food supplement) and medicines. Since the authors of the publication work in this field ^27^, we feel that this should be well known.

It is also common knowledge that lawful use depends on the definitions and legal provisions of the legislation enacted by the EU (in particular (EC) 178/2002 on the general principles of food law, and, for its delimitation, Directive 2001/83/EC ^28^ on medicinal products for human use) and the case law of the ECJ which completes and complements them. In addition, we have the Directive on Food Supplements 2002/46/EC in the EU, which explicitly permits food supplements with "nutritional as well as physiological effects". Most CBD products on the EU market fall into this category and are therefore not allowed to have a "pharmacological, immunological or metabolic action" (medicinal products by function pursuant to Medicinal Products Directive), and they are not allowed to claim any diagnostic, curative or therapeutic effect or to present themselves as such (the latter as so-called "medicinal products by presentation").

However, it is also undeniable that natural CBD in food or food supplements is useful and justified because of its physiological effects (in low doses far from those used medicinally) ^29^ – just like other natural substances – especially in the natural matrix of a hemp extract. Even the World Health Organization reports this in its comprehensive latest study from 2018 on Cannabis and THC ^30^.

Therefore, it is not clear why the above-mentioned legal principles and those developed by case law on their classification as food/ingredient should not apply to products containing hemp. The opinion and demand made by Lachenmeier *et al.* in their conclusions would ultimately mean a general ban on the current use and marketing of products containing hemp as food ingredients. But there is no plausible reason for this, and certainly no scientifically recognised reason.

## Summary

We conclude that the content of the publication by Lachenmeier *et al.* has fundamental flaws, and, in our opinion, does not meet the expectations of a scientific work that should be based on clear facts and evidence. Accordingly, we would suggest that due to its scientific shortcomings, as outlined above, it cannot be consulted and used without restriction for the objective and correct assessment of concrete facts.

Nevertheless, EIHA would like to confirm that unfortunately there are certainly a few products on the market that do not contain the level of cannabinoids stated on the label, or which make unjustified and unauthorised health claims on physiological effects. EIHA therefore supports the creation of clear industry standards that are mandatory for all food operators in this segment to ensure legal compliance and safety for both producers and consumers. By now many FBO's already submit themselves to a "self-regulated" quality standard, which is why we are currently trying to harmonise these standards as to scope and values – there are already some proposals on the table and work is in progress.

EIHA is working meticulously with the EU Commission and other industry groups on the concepts of a reliable quality assurance system for industrial hemp products. The corresponding proposals will be presented soon, after which they will be assessed by an EU legislator.

## Data availability

All data underlying the results are available as part of the article and no additional source data are required.

## Notes

^1^ “ *Are side effects of cannabidiol (CBD) products caused by tetrahydrocannabinol (THC) contamination?” b*y Lachenmeier et al. in the online Journal F1000Research 2020, 8:1394; Last updated 17 Feb 2020

^2^ Geffrey *et al.*: Drug-drug interaction between clobazam and cannabidiol in children with refractory epilepsy, Epilepsia 56(8): 1246–51, 2015

^3^ U.S. Public Health Service, Dep. of Health and Human Services, Office of the Secretary, Letter to the DEA, dated May 16, 2018; Enclosure: Basis for the recommendation to place Cannabidiol in Schedule V of the Controlled Substances Act., Chapter B.2, page 12 (Residual THC Levels)

^4^ FSA (2020). ‘Food Standards Agency Board meeting – 21 January 2020’. FSA, 3 January. https://www.food.gov.uk/sites/default/files/media/document/fsa-20-01-03-chief-executives-report-final.pdf

^5^ Dewey, W.L. 1986. Cannabinoid pharmacology. Pharmacol Rev 38(2):151–178;

G. Moreno-Sanz: Can You Pass the Acid Test ? Critical Review and Novel Therapeutic Perspectives of Δ ^9^-Tetrahydrocannabinolic Acid A, Cannabis Cannabinoid Res. 1.1 (2016).

^6^ https://eur-lex.europa.eu/legal-content/EN/TXT/?uri=CELEX%3A32016H2115

^7^ If it were total-THC, it would not allow any conclusions on the possible daily intake and thus on reaching or not reaching the LOAEL because the latter has been derived by EFSA on the basis of studies with Delta9-THC [Acid-free] only. Consequently, the Acute Reference Dose (ARfD) derived by EFSA in its scientific opinion only refers to Delta9-THC [acid-free].

^8^ Lachenmeier, D. W. (2020, April 26). Dataset for “Are side effects of cannabidiol (CBD) products caused by delta9-tetrahydrocannabinol (THC) contamination?” https://doi.org/10.17605/OSF.IO/F7ZXY

^9^ Hazekamp *et al.,* Cannbis tea revisited: A systematic evaluation of the cannabinoid composition of cannabis tea, J. Ethnopharmacol. 113 (2007) 85–90

^10^ Test reports are available on request from EIHA.

^11^ EFSA CONTAM Panel (EFSA Panel on Contaminants in the Food Chain), 2015. Scientific Opinion on the risks for human health related to the presence of tetrahydrocannabinol (THC) in milk and other food of animal origin. EFSA Journal 2015;13(6):4141, 125 pp. doi: 10.2903/j.efsa.2015.4141

^12^ Austria has no recommendations or limit values for maximum levels of THC in foodstuffs. There authorities only determine the conformity with the ARfD for Delta9-THC [acid-free] from the EFSA recommendation.

^13^ https://eur-lex.europa.eu/legal-content/EN/ALL/?uri=celex%3A32002L0046

^14^ Zuardi *et al*.: Action of Cannabidiol on the Anxiety and Other Effects Produced by Δ ^9^-THC in Normal Subjects, Psychopharmacol. 1982(76): 245-50.

^15^ Fischer *et al*.: Lower-Risk Cannabis Use Guidelines: A Comprehensive Update of Evidence and Recommendations, Am J Public Health. 2017 August; 107(8): e1–e12, Published online 2017 August. doi: 10.2105/AJPH.2017.303818.

^16^ Pisanti *et al*.: Cannabidiol: State of the Art and new challenges for therapeutic applications, Pharmacol. Ther., 2017 (175), 133–50.

^17^ https://ec.europa.eu/info/legal-notice_en

^18^ BGH GRUR 2015, 1140; https://dejure.org/dienste/vernetzung/rechtsprechung?Gericht=BGH&Datum=16.04.2015&Aktenzeichen=I%20ZR%2027/14

Hegele, Zeitschrift für das gesamte Lebensmittelrecht (ZLR), Germany, 2012, 317, 322 f.

^19^ BVerwG decision of 29 January 2010, 3 B 84/09

https://dejure.org/dienste/vernetzung/rechtsprechung?Gericht=BVerwG&Datum=29.01.2010&Aktenzeichen=3%20B%2084.09

^20^ BVerwG, judgment of 26 May 2009 - 3 C 5.09

https://dejure.org/dienste/vernetzung/rechtsprechung?Gericht=BVerwG&Datum=26.05.2009&Aktenzeichen=3%20C%205.09

^21^ https://eur-lex.europa.eu/legal-content/EN/ALL/?uri=celex%3A32002R0178

^22^ Letters from the EU Commission to SonnenHaus ÖKO-Handels GmbH of February 3, 1998, and to Alfredo Dupetit Natural Products of March 3, 1998; https://eiha.org//media/2019/05/EIHA-PRESS-NOTES-EXTRACTS.pdf (see page 4). Copies of the letter are provided on request.

Presentation by M. Reinders: Bringing hemp based products to market, 4 ^th^ Dec 2019, Cannabis Innovation Hub, London, see charts 16, 17.

^23^ Statement of the Federal Government in the German Bundestag of 25.07.2019, printed matter 19/11922, p.2.

http://dipbt.bundestag.de/extrakt/ba/WP19/2506/250681.html

^24^ Examples of such companies are known to the EIHA and discussed with the corresponding author.

^25^ https://eiha.org/2020/06/16/novel-food-pioneering-scientific-studies-commissioned-by-eiha/

https://canna-biz.legal/author/cannabizlegal/

https://hempindustrydaily.com/european-hemp-group-members-approve-plan-for-cbd-thc-studies/

^26^ https://www.newsweek.com/2019/09/06/cbd-oil-miracle-drug-science-1456629.html

^27^ Lachenmeier, Walch: Cannabidiol (CBD): a strong plea for mandatory pre‑marketing approval of food supplements, Journal of Consumer Protection and Food Safety, 

https://doi.org/10.1007/s00003-020-01281-2

^28^ https://ec.europa.eu/health/sites/health/files/files/eudralex/vol-1/dir_2001_83_consol_2012/dir_2001_83_cons_2012_en.pdf

^29^ See e.g. Australian Government, Department of Health, Therapeutic Goods Administration: Safety of low dose cannabidiol, Version 1.0, April 2020.

https://www.tga.gov.au/alert/review-safety-low-dose-cannabidiol.

S. Shannon *et al*. :Cannabidiol in Anxiety and Sleep: A Large Case Series, The Permanente Journal 2019;23:18-041, https://doi.org/10.7812/TPP/18-041

^30^ WHO Expert Committee on Drug Dependence, Fortieth Meeting, 4–7 June 2018: Cannabidiol (CBD), Critical Review Report: https://www.who.int/medicines/access/controlled-substances/CannabidiolCriticalReview.pdf
