# Supplementary material for: Comment on Lachenmeier et al (2020) “Are side effects of cannabidiol (CBD) products caused by tetrahydrocannabinol (THC) contamination?”: disputation on various points in the publication
Source: F1000Res. 2020 Aug 4;9:900. [Version 1] doi: 10.12688/f1000research.25354.1 (PMC7445557; doi:10.12688/f1000research.25354.1)
Supplement: Peer review history

# Peer review: Reviewer response for version 1

**DOI:** 10.5256/f1000research.27978.r68709

**Published:** 2020-08-24

**Authors:** Gianpaolo Grassi

**Affiliations:** 1 Research Centre for Industrial Crops, Council for Agricultural Research and Economics (CREA-CIN), Rovigo, Italy

One of the most abundant problems in the article by Lachenmeier *et al.* (2020) is confusion. Many of these topics involved are described by previous reviewers and very well by Kruse and Beitzke. I would like to point out the problem of the heterogeneity of the products sampled and tested. They are very different from each other and certainly produced by many companies, in very different conditions, using variable botanical raw materials, of European and non-European origin. The origin adds great confusion to the discussion because if we consider the European hemp varieties we should find in them no more than 0.2% THC and therefore its residues in oil and extracts should be affected by this condition. In non-European varieties we have often found the THC level over 1% (five times the European level) and obviously the THC contamination of oils and extracts will be higher than in products derived from European varieties. Raw materials derived from non-European varieties are widely used in Italy because they are cheaper than European or Italian hemp raw materials (seeds and dried leaves).

 In Italy, the Ministry of Health has received in previous years only two reports of side effects derived from hemp oil, without the addition of CBD. The reason was that the origin of the seed used to extract the oil was from China and was used as a supplement taken over a long period of time with high daily doses prescribed by the doctor to a child and a young woman.

 A large number of samples tested by Lachenmeier *et al.* (No. 12) are leaves and flowers used as tea. In this situation the natural cannabinoid content cannot be modified by purification so only the harvest time and the age of the leaves could have a relationship with the THC content which will always be present, but certainly less than 0.2%. Tea preparation involves a high temperature for extraction and this always causes the complete decarboxylation of THC and the activation of its psychotropic effect.

 When we consider the samples evaluated by Lachenmeier *et al.* in the article, they mostly derive from CBD extract which we could find obtained in two main ways: 1. Concentrated cannabinoids (full spectrum) and 2. Purified CBD (99% pure in crystalline form).

 The most trusted companies use pure CBD derived from crystals dissolved in vegetable oils (sesame, olive, sunflower or hemp seeds). In the case of hemp seed oil, THC contamination is certainly in the 1-5 ppm range and this concentration is admitted by many European countries (Germany, Italy). CBD supplement or foods derived from crystallized non-psychotropic cannabinoids (CBD and CBG) should be identified on the product label so that consumers can be properly informed and could identify a company that describes it correctly the origin of the Product.

 Many of the problems related to the "THC-like" side effect could be avoided if the origin of the product or standardization of production could include the block chain procedure or a consortium of manufacturers following a strict production protocol (e.g. see Canadian Manufacturers Test Pledge). It is a consequence of the lack of complete and clear rules and control by third parties (National Authority) because the hemp and cannabis market includes hundreds of products with many origins and the safety of the active ingredients must be guaranteed.

 The suggestions to the Authorities listed in Kruse and Beitzke's document have been underlined in many countries for years and years, but politics has little interest in regulating the laws and the cannabis market probably because the size of this market is too small or probably because it is interesting for some lobbies.

 However, in the paper by Lachenmeier *et al.* there is a lot of confusion and opinions that are outside the scientific task of such a publication. Confusion is very often the strategy used to limit the ability of consumers and public opinion to make their own choices. In extreme situations, lies add to the confusion and in the case of cannabis this has been done frequently. It should be time for Europe to consider the topic of cannabis correctly and could make an objective choice supported by scientific evidence where opinion or personal orientation as reported in the article by Lachenmeier *et al.* should be omitted.

Are arguments sufficiently supported by evidence from the published literature or by new data and results?

Yes

Is the conclusion balanced and justified on the basis of the presented arguments?

Yes

Is the rationale for commenting on the previous publication clearly described?

Yes

Are any opinions stated well-argued, clear and cogent?

Yes

Reviewer Expertise:

Plant breeding, hemp, cannabis, cannabinoids, foods, regulation, THC

I confirm that I have read this submission and believe that I have an appropriate level of expertise to confirm that it is of an acceptable scientific standard.

# Peer review: Reviewer response for version 1

**DOI:** 10.5256/f1000research.27978.r68710

**Published:** 2020-08-10

**Authors:** Gerhard Nahler

**Affiliations:** 1 CIS Clinical Investigation Support GmbH, Vienna, Austria

**21/08/2020: Editorial Note**

 Since the publication of the peer review report, it has been brought to the editorial team's attention that some Competing Interests had not been declared.  As part of F1000Research's editorial policies, reviewers are required to declare any competing interests, including financial competing interests, which may influence their peer review report.  We have since confirmed with Dr Nahler that they have the following competing interests, which have been added to their peer review report:

*Gerhard Nahler works as independent consultant. Among others, he is consultant of the non-profit NGO “ICANNA”, the EIHA and a number of pharmaceutical industries.*

 In order to understand the counterstatement of Kruse and Beitzke (2020) it is necessary to read the article of Lachenmeier *et al.* (2020 ^1^) first.

 1) The primary message of Kruse and Beitzke in their Abstract is that no proof for putative side effects/adverse effects caused by “CBD-products” is provided by Lachenmeier *et al. *(2020), nor that such side effects are caused by THC.

 2) The second point is a lack of clarity about the term “THC” used by Lachenmeier *et al.* Is it used exclusively for the psychotomimetic delta-9-tetrahydrocannabinol (?), the non-psychotomimetic delta-9-tetrahydrocannabinolic acid (?), for a mixture of both substances such as occurring in hemp tea (?), or also for an analogue, delta-8-tetrahydrocannabinol (?) which is a minor byproduct with lower psychotomimetic properties in hemp extracts?

 3) The third point concerns the “safety” of commercial CBD-products analysed by Lachenmeier *et al.*, in relation to the content of THC. Here, Kruse and Beitzke criticise unrealistic assumptions concerning the calculation of the supposed daily intake of CBD-products and a lack of clarity of the table 2 in the main part of Lachenmeier’s article (concentrations of cannabinoids are communicated by Lachenmeier *et al.* only as a separate supplemental material; without, the reader cannot verify the assumptions of Lachenmeier *et al.*).

 Ad 1) According to the title, the article of Lachenmeier *et al.* (2020) focuses on “ ***side effects of*** ***cannabidiol (CBD) products***” caused by ” ***contamination***” with THC. As CBD products vary widely in their composition beyond CBD as main ingredient, a more detailed characterisation of products, their composition and various other phytocompounds, as well as a comparison to the characteristic side effects of the two pure substances, CBD and delta9-tetrahydrocannabinol (in short THC) would help to better understand the differences. In Lachenmeier’s article, various CBD-products based on hemp extracts or flowers, often consumed as “supplements”, are addressed, therefore containing not only CBD but a number of other phytosubstances, such as delta9-tetrahydrocannabinol (in short THC) among numerous other natural byproducts in varying ratios. The nature and quantities of these other byproducts depend, among many others, on the extraction process and may also include the respective acids CBDA and THCA which have different properties compared to the decarboxylated substances CBD and THC. Lachenmeier’s article focuses therefore on ill characterised “CBD-products”. As a uniform “CBD-product” does not exist, products likely vary in their activity and side effect profile. The statement of Lachenmeier *et al.* on page 6 “ *our* *results provide compelling evidence that THC natively contained in CBD products by contamination may be a direct cause for side effects of these products*” is not supported by a listing of such side effects, including their frequencies and/or the respective literature. The title of Lachenmeier’s article assumes that side effects occur with CBD-products and the reader might expect that this will be further addressed in the article; however, this is not the case. The only “side effects” reported read as follows (section “Introduction”): “ *some pediatric studies in epilepsy patients with orally administered CBD also reported adverse effects such as drowsiness and fatigue that could be explained by pharmacological properties of THC rather than of CBD (8–10*).” A further, more detailed description of the nature of side effects supposed to be caused by THC in CBD-(food) products is missing. This is clearly criticised by Kruse and Beitzke. Patients in the articles referenced by Lachenmeier *et al.* received either extracts (Ref.8, a US parent-survey) or pure CBD (CBD/Epidyolex of GW, purity ~99% with ≤0.1% THC) for treatment-resistant epilepsy and in daily dosages exceeding in some cases 25mg CBD/kg body weight. These subjects received, however, antiepileptic drugs in addition, known to cause, e.g., sedation. Therefore, this can hardly serve as reference for side effects of CBD-products (notably food-products, hemp extracts) taken by a normal, healthy population as is addressed by Kruse and Beitzke. Indisputable differences are therefore the product itself (pure CBD vs. mixtures of phytocompounds), the population (patients vs. healthy individuals) and the intake (regular, high concomitant therapeutic doses vs. low dose, often taken occasionally).

 Although this would be of considerable interest, the reviewer is unaware of any systematic evaluation of side effects supposed to be related to CBD-containing, hemp-derived food products. On the contrary, CBD has been described to reduce side effects of THC as has been commented by Kruse and Beitzke. In the article cited (Zuardi *et al.*, 1982 ^2^) a mixture of CBD with THC (ratio 2:1) attenuated anxiety reactions caused by THC. In hemp, the ratio of CBD:THC is much higher, typically around 20:1; such a modified ratio may also have modified effects which can be further influenced by other phytocompounds. Pure CBD seems to be safe; after excluding studies in childhood epilepsy, the only adverse outcome associated with CBD treatment was diarrhoea (Chesney *et al.*, 2020 ^3^). In the EFSA report (EFSA, 2015 ^4^) the lowest observed adverse effect level (LOAEL) of 2.5 mg Δ9-THC/day, corresponding to 0.036 mg Δ9-THC/kg b.w. per day, was derived from “ *observed central nervous system (CNS) effects* (i.e. slight euphoric effects), *as well as the increase in heart rate*” [according to a more recent review, “a *dose of 7.5 mg *(THC) *did not affect heart rate or blood pressure*” (WHO, 2018 ^5^)]. It has to be reminded that the effect of THC on the heart rate is dose-dependent and transient, and disappears after repeated administration. Whether a slight euphoric effect is “ *adverse*” or not may be disputable and still depends on the situation. Drowsiness or fatigue is uncommon with very low doses of THC.

 Ad 2) Although not explicitly stated, “THC” is the psychotomimetic delta-9-tetrahydrocannabinol. If the term is used for “mixtures” this may cause misunderstandings. This may have been the case when Lachenmeier *et al.* refer to hemp tea, even if it is a common analytical procedure to exhibit the sum of **THC + THCA as “THC” for forensic** reasons. In addition to request a discriminate use of the term “THC”, Kruse and Beitzke address also in their counterstatement the aspect of an appropriate “labelling” and of mandatory “industry standards” for CBD-products in order to limit a negative impact by “black sheeps” on the reputation of the whole hemp industry. This is highly welcome in the interest of the scientific community, and would be a considerable step forward as it makes discreditation of CBD-products and of the hemp industry less likely; this is clearly encouraged by Kruse and Beitzke. Although not explicitly mentioned as such by Kruse and Beitzke, it is obvious that a correct declaration of the content should not be restricted to the recommended daily consumption and the amount of CBD.

 Ad 3) In table 2 of Lachenmeier’s article, all samples for which " *THC > LOAEL*“ are marked in red (17 out of 40 samples); of these 17, seven samples*) (41%) are “hemp teas”. As this is a notable percentage it deserved a closer look by Kruse and Beitzke. The calculation of the THC-exposure after consumption of tea made from hemp flowers and/or leaves by Lachenmeier *et al.* is misleading: First, cannabinoids are almost water-insoluble, therefore an extraction of the total amount of THC contained in 8g of dry herbal substance by hot water – as has been assumed in Lachenmeier’s article - is impossible. Even when drug-type cannabis was used for preparation of a tea, the concentration after 20 minutes of boiling (which is not common practice for preparing tea) was 0,043 mg THCA/mL und 0,01mg THC/mL (Hazekamp *et al.*, 2007 ^6^) or 1.0 to 2.4mg THC per liter according to others (Giroud *et al.*, 1997 ^7^, cited by Hazekamp *et al.*, 2007). Consumption of two cups of tea, prepared as usual by pouring hot water on dry herbal substance and simmering for about 10 minutes, would normally not result in an uptake of THC exceeding the amount of the lowest observed adverse effect level (LOAEL). This is an important objection made by Kruse and Beitzke. The second, minor overestimation concerns the amount of 8g of dry herbal substance consumed per day. Regular tea bags usually weigh 1.5-2 grams; 8g correspond to 4 to 5 cups of tea per day; this seems to be a rather high consumption, above the average. (The exposure by other CBD-products than hemp tea has not been reviewed).

 When Lachenmeier *et al.* use, in addition to “ *side effects*”, the term “ ***unsafe***” for CBD-(food-) products, Kruse and Beitzke request, for the sake of clarity, an explanation why they are “unsafe”, i.e., a description of any, putative or confirmed, short- or long-term negative impact of CBD-products on human health. “Safe” or “unsafe” for food commonly implicates a broader impact on human health than (isolated) side effects. Food can induce, as an example, allergic reactions as side effects still being safe for the large majority of consumers.

 In short, Kruse and Beitzke clearly describe a number of points in Lachenmeier’s article which would deserve clarification.

 *)

 190267605

 180630663

190595270-tea

 180776480

190490183-tea

190595273-tea

190595267-tea

 190203194

 180598182

190495001-tea

 190203193

 180781746

190400870-tea

 180198245

 180198246

 180598187

190176314-tea

**( 7** **of 17 samples); **in table 2 of Lachenmeier’s article all samples for which „ *THC > LOAEL*“ are marked in red (17 out of 40 samples); of these 17, seven samples (41%) are “hemp teas”.

Are arguments sufficiently supported by evidence from the published literature or by new data and results?

Yes

Is the conclusion balanced and justified on the basis of the presented arguments?

Yes

Is the rationale for commenting on the previous publication clearly described?

Yes

Are any opinions stated well-argued, clear and cogent?

Yes

Reviewer Expertise:

NA

I confirm that I have read this submission and believe that I have an appropriate level of expertise to confirm that it is of an acceptable scientific standard.

## References

[1] : Are side effects of cannabidiol (CBD) products caused by tetrahydrocannabinol (THC) contamination?. *F1000Res*.2019;8: 10.12688/f1000research.19931.2 1394 10.12688/f1000research.19931.2 32117565PMC7029751

[2] : Action of cannabidiol on the anxiety and other effects produced by delta 9-THC in normal subjects. *Psychopharmacology (Berl)*.1982;76(3) : 10.1007/BF00432554 245-50 10.1007/BF00432554 6285406

[3] : Adverse effects of cannabidiol: a systematic review and meta-analysis of randomized clinical trials. *Neuropsychopharmacology*.2020; 10.1038/s41386-020-0667-2 10.1038/s41386-020-0667-2 32268347PMC7608221

[4] : Scientific Opinion on the risks for human health related to the presence of tetrahydrocannabinol (THC) in milk and other food of animal origin. *EFSA Journal*.2015;13(6) : 10.2903/j.efsa.2015.4141 10.2903/j.efsa.2015.4141

[5] WHO 2018 Expert Committee on Drug Dependence Pre-Review: Delta-9-tetrahydrocannabinol. Section 3: Toxicology. *World Health Organization*.2018; Reference source

[6] : Cannabis tea revisited: a systematic evaluation of the cannabinoid composition of cannabis tea. *J Ethnopharmacol*.2007;113(1) : 10.1016/j.jep.2007.05.019 85-90 10.1016/j.jep.2007.05.019 17604926

[7] : Hemp tea versus hemp milk: subjective effects and elimination studies of THC and its main metabolite. *Proceedings of the 35th TIAFT meeting, Padova, Italy*.1997;112-121
